# Carboxymethyl cellulose coated magnetic nanoparticles transport across a human lung microvascular endothelial cell model of the blood–brain barrier

**DOI:** 10.1039/c8na00010g

**Published:** 2018-10-16

**Authors:** Gabriela Aguilera, Catherine C. Berry, Rachel M. West, Enrique Gonzalez-Monterrubio, Aracely Angulo-Molina, Óscar Arias-Carrión, Miguel Ángel Méndez-Rojas

**Affiliations:** Departamento de Ciencias Químico-Biológicas, Universidad de las Américas Puebla Ex Hda. Sta. Catarina Mártir s/n, San Andrés Cholula 72820 Puebla Mexico miguela.mendez@udlap.mx +52 222 2292416 +52 222 2292607; Centre for Cell Engineering, Institute of Molecular Cell and Systems Biology, University of Glasgow Joseph Black Building, University Avenue Glasgow Scotland; Unidad de Trastornos del Movimiento y Sueño (TMS), Hospital General Dr Manuel Gea González Av. Calzada de Tlalpan 4800, Col. Sección XVI C. P. 14080 Mexico City Mexico arias@ciencias.unam.mx +52 55 52115199 +52 155 26849064; Departamento de Ciencias Químico-Biológicas/DIFUS, Universidad de Sonora Luis Encinas y Rosales, s/n Colonia Centro, 83000 Hermosillo Sonora Mexico

## Abstract

Sustained and safe delivery of therapeutic agents across the blood–brain barrier (BBB) is one of the major challenges for the treatment of neurological disorders as this barrier limits the ability of most drug molecules to reach the brain. Targeted delivery of the drugs used to treat these disorders could potentially offer a considerable reduction of the common side effects of their treatment. The preparation and characterization of carboxymethyl cellulose (CMC) coated magnetic nanoparticles (Fe_3_O_4_@CMC) is reported as an alternative that meets the need for novel therapies capable of crossing the BBB. *In vitro* assays were used to evaluate the ability of these polysaccharide coated biocompatible, water-soluble, magnetic nanoparticles to deliver drug therapy across a model of the BBB. As a drug model, dopamine hydrochloride loading and release profiles in physiological solution were determined using UV-Vis spectroscopy. Cell viability tests in Human Lung Microvascular Endothelial (HLMVE) cell cultures showed no significant cell death, morphological changes or alterations in mitochondrial function after 24 and 48 h of exposure to the nanoparticles. Evidence of nanoparticle interactions and nanoparticle uptake by the cell membrane was obtained by electron microscopy (SEM and TEM) analyses. Permeability through a BBB model (the transwell assay) was evaluated to assess the ability of Fe_3_O_4_@CMC nanoparticles to be transported across a densely packed HLMVE cell barrier. The results suggest that these nanoparticles can be useful drug transport and release systems for the design of novel pharmaceutical agents for brain therapy.

## Abstract 2

CMC coated magnetic nanoparticles cross through a densely packed Human Lung Microvascular Endothelial (HLMVE) cell barrier BBB model.

## Graphical abstract

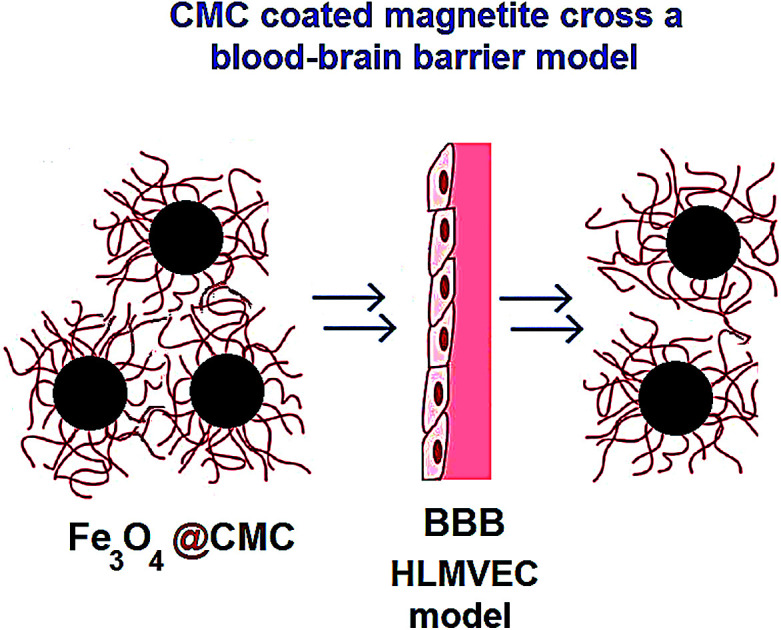

## Introduction

Neurodegenerative disorders such as Parkinson's and Alzheimer's diseases are the leading cause of disability and the second cause of mortality worldwide; these disorders now affect more than 250 million people globally. This number is expected to rise substantially as current population growth and the increase in life expectancy mean that more people will reach the age ranges where these disorders are prevalent.^1^ At present, no cure is available for CNS disorders and they are difficult to treat pharmacologically, at best their treatment is palliative.^2^ Low bioavailability, adverse reactions, and inefficient targeting of the brain are just a few of the disadvantages of the pharmacological therapies currently in use. Consequently, there is a pressing need to develop more efficient non-evasive and brain-directed therapies for neurological disorders.

While brain-targeted drug delivery has been gaining increasing attention, these strategies pose a significant challenge for drug developers. Due to the very high cost of their development and the potential for undesired side effects and long-term health risks, new pharmaceutical formulations for the treatment of neurological disorders have the lowest approval rate in the drug development pipeline.^3^ Development of drugs for the CNS is extremely costly. Only 3–5% of the pharmaceuticals intended for brain delivery reach the market, as most are unable to cross the BBB *in vivo*.^4,5^ Although it is well known that larger molecules (∼100%) have restricted BBB permeability, most small molecules (>98%) are also unable to cross the BBB. An estimated 95% of the small molecules in drug discovery libraries have very limited ability to permeate the BBB, tempering their pharmacological usefulness for the treatment of neurological disorders.^4^

Successful development of CNS drugs requires an understanding of both the pharmacological target and achieving sufficient permeability across the BBB to attain effective therapeutic concentrations in the brain. The functional complexity of this barrier demands the development of different strategies to effectively overcome it. One such strategy involves the design of more efficient drug delivery systems that can transport promising therapeutic and/or imaging agents over the BBB.^6^ Therefore, research in the field of nanotechnology has started to focus on the generation of nanostructured drug delivery carriers capable of crossing the BBB and delivering drugs to specific sites in the brain. Nanoscale drug delivery devices can absorb and carry drugs and then due to their ultra-tiny volume they can pass through the smallest capillary vessels to penetrate cells and tissue gaps. Their size allows them to avoid rapid RES clearance, so their duration in the bloodstream is greatly extended. The increased safety, efficacy, and bioavailability of nanoparticles (NPs) make them attractive options in the investigation of pharmacological therapies for neurological disorders.^7^

Targeted delivery of these systems significantly reduces the required dosage, which may decrease the undesired effects of the treatment of these disorders such as adverse reactions and toxicity.^8^ Although brain targeting delivery systems can enhance the distribution of therapeutic drugs in the brain, an important factor to consider in the design of these systems is nanoparticle accumulation. As the treatment of chronic neurological disorders often requires long-term and frequent drug administration, nanoparticles could potentially build up in the body, causing undesirable side effects. To provide biological safety, the materials used for these systems should be biodegradable, compatible with the metabolic system, able to be eliminated from the brain and have a high potential for biological and biomimetic effects. Recently, research has focused on the use of natural materials for the fabrication of nanocarriers, as they inherently possess many of these qualities.

Magnetite (Fe_3_O_4_) SPIONs have received widespread acceptance within the scientific community as their magnetic properties make them highly attractive for biomedical applications, especially as agents for MRI and targeted drug delivery.^9,10^ The biocompatibility of these particles is frequently enhanced with organic or inorganic coatings that allow their suspension in aqueous or organic media.^11^ After drug molecules are attached to these delivery systems, the biodegradability, pH, ion and/or temperature sensibility of the materials can be used to activate drug delivery at a controlled and sustained rate to the target areas of the brain.^12^ An advantage of biopolymer-coated magnetic nanoparticles is that they have shown lower toxicity levels compared to those with bare cores. This phenomenon is attributed primarily to the spontaneous aggregation of the bare cores induced by the presence of plasmatic proteins and salts in biological media. The addition of polymeric layers confers an “anti-aggregation” barrier to the magnetic cores, as well as an appropriate surface for functionalization.^13^

The first nanocarriers were coated with artificial^14–17^ or inorganic^16,18–21^ polymers. Poly(ethylene glycol) (PEG) was often attached to the surface of these NPs as a ‘‘stealth layer’’ to decrease protein adsorption and to increase their concentration in the *in vivo* circulation.^16–18,22^ However, there were a number of drawbacks to these particles, particularly concerning their biocompatibility and biodegradability, which may result in adverse side effects, whereas, natural polymers are proving to be a very attractive option for coating NPs as their chemical similarity to biomolecules already present in extracellular matrices affords the resulting nanomaterial high bioactivity and biocompatibility.

The contemporary nanoparticle drug delivery field is studying several of these natural polymers as alternatives to PEG. Most of this work has focused on chitosan-based materials,^23–26^ while comparatively little work has been done with other polysaccharides. Polysaccharides have been reported to reduce unspecific protein adsorption and increase plasmatic life.^27^ Furthermore, the biodegradability of these materials not only facilitates the eventual clearance of the nanocarrier but can be exploited to trigger drug release and activation by using certain enzymes to produce controlled degradation of the coating.^28–31^ These properties, together with their ability to interact with certain protein/cell surfaces and reduce particle aggregation make polysaccharides very interesting materials for the construction of brain-targeted NPs.

Carboxymethyl cellulose (CMC) is produced from the reaction of cellulose (from wood pulp or cotton fibers) under basic conditions with chloroacetic acid.^32^ Although this biopolymer is well known and highly versatile, successful uses of CMC for biomedical applications of nanoparticle technologies are quite limited. CMC can be used for the modification of magnetite nanoparticles as the high density of carboxylate ions per chain allow it to be physically adsorbed or conjugated on the surface of the nanoparticles, yielding a highly stable, water-soluble colloidal solution, and it is easy to modify with a reporter or targeting species.^33–38^ Studies exploring brain delivery systems indicate that nanoparticle surface charge has an important role in determining cellular uptake and the interactions between particles and cells.^39^ The negative charge of the CMC coating not only has a favorable impact on the nanoparticles' colloidal stability,^39,40^ it favors less plasma protein adsorption and therefore increases the plasma circulation time of the particles and confers other properties that make CMC a suitable coating material for *in vitro* and *in vivo* applications in the investigation of brain-targeted magnetic nanoparticles.^41^

In the past decade, enhanced properties such as adhesion of ligands (antibodies, proteins or other systems) on the NP surface, ligand density, and NP shape have been shown to improve the transport of NP formulations through the BBB as well as improving molecular recognition and controlled release in specific targets.^42–45^ Although these advances have become very popular, there are still a few important drawbacks to their use, such as drug-release failure resulting from the high stability of the generated bonds or reduced circulation times of the NPs due to alteration of the physicochemical properties when the ligands are attached.^46^ These drawbacks offset the potential benefits of active targeting as they affect the bioavailability and specific molecular recognition ability of the NPs.^47^

Our group has been exploring the biomedical applications of magnetic nanoparticles as drug carriers, MRI contrast agents and for magnetic hyperthermia.^48–53^ Most recently our research has focused on exploring alternative biocompatible polymeric coatings for these nanoparticles.^54,55^ Here, the preparation and characterization of CMC coated magnetite nanoparticles (Fe_3_O_4_@CMC) and the evaluation of their performance as drug delivery systems for dopamine are presented. Fluorescence and electron microscopy (SEM and TEM) studies were used to analyze the cell viability of HLMVE cell cultures exposed to the magnetic nanoparticles and their interaction with cell components. The ability of these CMC coated SPIONs to move through a BBB model was determined using the transwell assay. The results of our *in vitro* assays suggest that these systems may be useful for drug delivery to the brain. Furthermore, the simplicity of preparation, plentiful supply and the safety and biocompatibility of the components of these CMC coated magnetite NPs could offer a considerable reduction in the cost of the developmental phase of brain-targeted pharmaceuticals using these delivery systems.

## Experimental

### Materials

Analytical grade FeCl_3_·6H_2_O, NH_4_OH, Na_2_SO_3_, 3-aminopropyl-trimethoxysilane (APTMS), carboxymethyl cellulose (CMC) and fluorescein were purchased from Sigma-Aldrich; fluorescent dyes 4,6-diamidino-2-phenylindole (DAPI) (Molecular Probes Inc; Eugene, OR, USA) and rhodamine phalloidin (Cytoskeleton Inc.; Denver, CO, USA) were used as received without further purification. Water was doubly deionized, rendering conductivity in the range of 16–18 MΩ. Stock 2 M aqueous solutions of FeCl_3_ (dissolved in HCl 2 M) and Na_2_SO_3_ were freshly prepared. Glassware was cleaned with concentrated HCl, rinsed with deionized water and dried before use.

### Synthesis of magnetite NPs (Fe_3_O_4_)

Magnetite (Fe_3_O_4_) nanoparticles were prepared based on a previously reported method.^48,56^ In summary, 1.08 g of FeCl_3_ was dispersed in 10 mL of DI water and stirred at 500 rpm until complete dissolution. Separately, 0.3975 g of FeCl_2_ was dissolved in 10 mL of DI water and stirred at 500 rpm until complete dissolution. Then, the Fe(ii) solution was added rapidly to the Fe(iii) solution while stirring strongly, and immediately afterward 2.5 mL of NH_4_OH (30%) was added. A black suspension formed and the recovered black solid was washed several times with deionized water. Finally, the nanoparticles produced with this method were separated by centrifugation, vacuum-dried at room temperature and stored. The nanoparticle size, as determined by TEM, was in the range of 11–17 nm. A strong tendency to agglomerate was observed in these nanoparticles. DLS analysis of the uncoated magnetic nanoparticles in DI water determined the average hydrodynamic diameter to be 30 nm.

### Preparation of fluorescein-labeled silanized magnetite NPs (fSi–Fe_3_O_4_)

100 mg of the previously prepared magnetite was ground in an agate mortar with anhydrous toluene (3 mL) until a fine powder was obtained. Then, the powder was transferred to a 250 mL round bottom flask and dispersed in 57 mL of anhydrous toluene. A volume of 20 μL of 3-aminopropyltrimethoxysilane (APTMS) was added to this black suspension and stirred at 60 °C for 4 hours. Finally, the magnetic precipitate was magnetically decanted, washed twice with absolute ethanol, and then vacuum-dried at 50 °C for 30 minutes. Labeling with fluorescein was achieved following the well-known EDC/NHS coupling protocol of adding fluorescein (acid form) during silanization of the magnetite nanoparticles in a mixture of water/DMF.^57^

### Coating with carboxymethyl cellulose (preparation of Fe_3_O_4_@CMC and Si–Fe_3_O_4_@CMC)

Both pure Fe_3_O_4_ and Si–Fe_3_O_4_ were coated with CMC following the same procedure. 100 mg of dried, finely ground magnetic nanoparticles were dispersed in 10 mL of DI and sonicated for 10 min. An aqueous 0.5% solution of sodium carboxymethyl cellulose (NaCMC) was prepared by dissolving 10 mg of NaCMC in 10 mL of DI, mechanically stirring until complete dissolution and then adding the NaCMC solution dropwise to the magnetic nanoparticle suspension and stirring at 500 rpm for 10 h. After this time, the material was magnetically decanted and washed once with DI. It was then dried in a vacuum oven at 50 °C for 20 min which yielded a dark brown powder. DLS analysis of CMC coated magnetite in aqueous suspensions shows hydrodynamic radii in the range from 40 to 120 nm, depending on the coating time, with zeta potential values (*ζ*) of −50 to 70 mV.

### Characterization

Fourier Transform Infrared (FT-IR) spectra of the nanostructured materials were recorded using a Varian Scimitar FTIR spectrophotometer equipped with an ATR detector and recorded in the region of 3000–600 cm^−1^. Dynamic light scattering (DLS) and zeta potential (*ζ*) measurements were performed using a Nanotrac Wave II (Microtrac) instrument, working at 28 °C in DI water as the dispersing medium, with a red laser of 780 nm, 3 mW. The crystalline phase of the iron oxide nanoparticles was identified by powder X-ray diffraction (XRD). The patterns were collected between 20 and 70° (2*θ*) using a Bruker-AXS D5000 diffractometer on ground powders in a quartz sample holder using the Cu Kα line source (*λ* = 1.5418 Å); step scan = 0.02; step time = 0.6 s. Scanning electron microscopy (SEM) images were obtained using a Tescan VEGA-II microscope, with an accelerating voltage of 20 kV. SEM specimens were dispersed in ethanol by ultrasonication, and a few drops were deposited on a graphite film adhered to an Al pin. The size and morphology of the NP were determined by transmission electron microscopy (TEM) using a JEOL JEM-120EXII electron microscope. TEM samples were prepared by placing one drop of a dilute suspension of magnetic nanoparticles in water on a carbon-coated copper grid and allowing the solvent to evaporate at room temperature. The average particle size was evaluated by measuring the largest internal dimension of ∼200 particles. High-resolution transmission electron microscopy (HRTEM) analyses were performed using a JEOL Model JEM2010 electron microscope operated at 200 kV accelerating voltage.

### Cell culture and nanoparticle internalization

HLMVE cells, obtained from the Centre for Cell Engineering at the University of Glasgow, were grown to confluence in Dulbecco's modified Eagle's medium (DMEM) with 50 units mL^−1^ penicillin, 50 mg mL^−1^ streptomycin, and supplemented with 5% fetal bovine serum (FBS), at a final concentration of 10%. All the media, serum, and antibiotics were provided by Life Technologies (Life Technologies, UK). Cell cultures were performed in a 5% CO_2_ atmosphere at 37 °C and maintained in an incubator. For the experiments, the cells were seeded into 24-well plates at an initial density of 1 × 10^4^ cells per well. Treatments were initiated three days after plating (approximately 70% confluence). For certain experiments (SEM and TEM analyses, and live/dead cell detection), the cells were seeded on 13 mm square glass coverslips placed into the wells. To analyze the internalization of nanoparticles, HLMVE cells were grown on coverslips and incubated for 24 h with different concentrations of nanoparticles (0.01, 0.1 and 1.0 mg mL^−1^).

For SEM analysis, after incubation, the containing medium was removed; the cells were washed three times with PBS and fixed with 1 mL of 1.5% glutaraldehyde in cacodylate buffer and 2% sucrose at 4 °C for 10 min. The cells were osmicated first with 1% osmium tetroxide, and then with 2% uranyl acetate for 5 min. After this, each slide was transferred into a Petri dish containing hexamethyldisiloxane (HDMS), dried in a desiccator and sputter-coated with a thin layer of gold in preparation for scanning electron microscopy (SEM) analysis. For TEM analysis, 13 mm Thermanox coverslips for seeding cells were used in 24-well plates; cell fixation was carried out following the previously described procedure. The samples were then dehydrated and embedded in EPON-812 resin, then frozen in liquid nitrogen and sectioned using an ultramicrotome. Slices were mounted on copper grids and analyzed by transmission electron microscopy.

### Live/dead cell detection using fluorescence microscopy

The cells were seeded on 13 mm square glass coverslips, placed into 24-well plates and incubated for 24 h. Then, the medium was replaced with fresh medium containing nanoparticles and incubated for 24 h, at 37 °C and 5% CO_2_. Next, the wells were washed with Ham's F-10 medium and 1 mL of staining solution (2 μM calcein AM and 2 μM ethidium homodimer-1) was added to each well and incubated for 1 h at 37 °C. For each sample, the assay was performed in duplicate. The cytoskeleton and cell nuclei were stained with rhodamine phalloidin (200 μL) and DAPI (50 μL), following standard procedures.^58,59^ Briefly, the cells were fixed in glutaraldehyde and, permeabilized (100 mL PBS; 10.3 g sucrose; 0.292 g NaCl; 0.06 g MgCl_2_ (hexahydrate); 0.476 g HEPES and pH adjusted to 7.2, followed by the addition of 0.5 mL Triton X). Non-specific binding sites were blocked by incubation with PBS/1% BSA for 5 minutes at 37 °C prior to incubation with rhodamine phalloidin for 1 hour at 37 °C (Thermo Fisher Scientific, Alexa Fluor™ 488 Phalloidin). Following washing, the cells were further incubated with DAPI mounting medium (Vector Laboratories). All images were viewed using an Axiophot fluorescence microscope.

### Evaluation of cell viability

The viability of the HLMVE cells was determined using a standard methylthiazol tetrazolium bromide (MTT) assay. Briefly, this involved incubation of the cells with unloaded or dopamine-loaded nanoparticles in 24-well plates for 24 and 48 h, after which MTT was added to each well (the final concentration of MTT was 5 mg mL^−1^ in PBS) for 1.5 h at 37 °C and 5% CO_2_. Then 100 μL of DMSO was added to each dish to dissolve the formazan crystals that formed in the cells. The absorbance was measured with a microplate reader (Tecan Spectra Fluor spectrophotometer) at 570 nm for each well. Cell survival was determined by the percentage of absorption of treated cells in comparison with that of control cells (incubated without nanoparticles) and was calculated using the following equation:% Cell viability = (absorbance of sample well/absorbance of control well) × 100

The results are the mean value and standard deviation (SD) obtained from three repetitions of the experiment (*n* = 3). The total amount of dopamine loaded onto the nanoparticles used for cell exposure never exceeds 0.13 mg per mg of magnetite.

### Transwell migration assay

Cell culture inserts (transwells) with a density of 1 × 10^5^ HLVMEC cells per well were overlaid with 125 μg mL^−1^ growth factor-reduced Matrigel (diluted in HamF10) and placed in a 24-well plate, at 37 °C and 5% CO_2_, and incubated for 24 h. For each sample, the assay was performed in duplicate. After incubation, the medium was replaced with fresh medium containing magnetic nanoparticles labeled with fluorescein (fSi–Fe_3_O_4_), at a concentration of 1 mg mL^−1^. To evaluate the integrity of the monolayer as a BBB model the transendothelial electrical resistance (TEER) was measured using an EVOM2 epithelial voltmeter with an STX2 electrode (World Precision Instruments) at 12.5 Hz, as reported previously.^60^ These measurements were taken during three stages of the test: before addition of the magnetic nanoparticles, and after 24 and 48 h of incubation with them. After exposure to the magnetic nanoparticles, the culture medium in the bottom of the well was recovered and transferred to a tube and pelleted by centrifugation at 1200 rpm for 3 min. The supernatant was eliminated, and 30 μL of the pellet was transferred to a glass slide for analysis by fluorescence microscopy.

### Dopamine loading and release studies

To determine the performance of the NPs as drug carrier and release systems, dopamine hydrochloride (Aldrich) was dissolved in DI water and then diluted to obtain concentrations within the range of 1 to 10 mg mL^−1^. A UV-visible spectrophotometer was used to determine the absorbance of the above concentrations at 280 nm using DI water as a blank. These concentrations were used to plot a calibration curve. The tests were carried out at room temperature (25 °C) and pH 7.0. For loading the nanocarrier, 10 mg of dried CMC coated magnetite nanoparticles (Fe_3_O_4_@CMC), prepared as previously indicated, were dispersed in 50 mL of an aqueous solution containing dopamine (30 μg mL^−1^) at pH 7.0, and mechanically stirred at 200 rpm for 10 h. After this time, the nanoparticles were magnetically decanted and washed with ice-cold water, and then dried for 2 h in a vacuum oven at 50 °C. The amount of dopamine loaded onto the nanoparticles was determined spectrophotometrically (at *λ* = 280 nm) by measuring 1 mL of aliquots at spaced intervals (0, 5, 10, 30, 60, 180, 540, and 600 min) during the incubation time. The amount of dopamine entrapped within the nanoparticles was calculated from the difference between the total amount of dopamine (*M*_1_) used to prepare the nanoparticles and the amount of dopamine present in the aqueous phase (*M*_2_). The following formula was used:
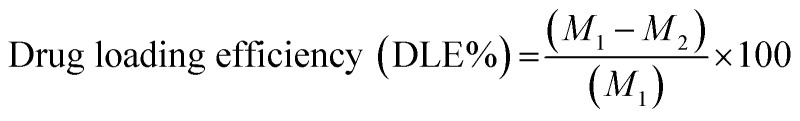


To determine dopamine release kinetics, 10 mg of the dried drug-loaded nanoparticles were dispersed in 50 mL of DI water at room temperature and pH 7. The concentration of free dopamine in the aqueous solution was determined using a UV spectrophotometer under constant mechanical stirring (200 rpm), for 4 h. 1 mL aliquots were taken at different time intervals (0, 5, 10, 30, 60, 180, and 240); the amount of released dopamine was determined using the previously established calibration curve.

## Results and discussion

### Synthesis and characterization of magnetic nanoparticles

**Fig. 1:**
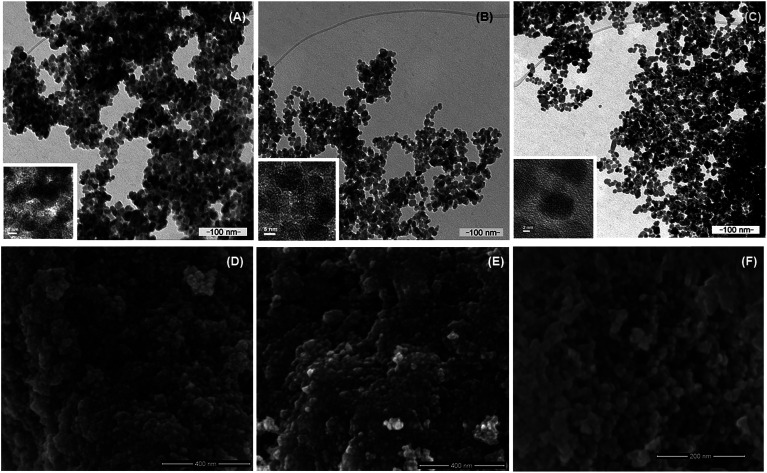
Transmission (TEM) and scanning (SEM) electron microscopy images of magnetic nanoparticles: (A) and (D) Fe_3_O_4_ (14.38 ± 3.06 nm); (B) and (E) Fe_3_O_4_@CMC (14.05 ± 1.70 nm); (C) and (F) Si–Fe_3_O_4_@CMC (14.96 ± 4.16 nm). Insets are HRTEM micrographs for the corresponding samples.

Nanoparticles of magnetite, Fe_3_O_4_, and silanized magnetite, Si–Fe_3_O_4_, were synthesized as superparamagnetic carrier cores through the chemical coprecipitation method.^56,61^ These magnetic nanoparticles were coated with the polysaccharide carboxymethyl cellulose (CMC) by an electrostatic adsorption method.^62^ Silanization of the magnetite nanoparticles was tested to ascertain if it improved the stability of the NPs as well as the immobilization of CMC on the nanoparticle surface. No significative differences were found between the non-silanized and silanized CMC-coated nanoparticles. The hydrodynamic radii were similar in both cases (around 110.0 ± 10.0 nm), and the aqueous suspensions were stable for several days at room temperature, which agrees with their highly negative zeta potential values. The morphology and size distribution of the nanomaterials were characterized by SEM and TEM. Fig. 1 shows selected representative scanning (SEM) and transmission electron microscopy (TEM) images of the different magnetic nanoparticles in this work (Fe_3_O_4_, Fe_3_O_4_@CMC, and Si–Fe_3_O_4_@CMC nanoparticles); the particles are mostly spherical and show a narrow size distribution, with average sizes of 19.90 nm ± 3.06 nm (Fe_3_O_4_), 14.05 ± 1.70 nm (Fe_3_O_4_@CMC) and 14.96 ± 4.16 nm (Si–Fe_3_O_4_@CMC). Smaller nanoparticle average sizes were obtained when the nanoparticles were coated with CMC, which may be the result of reduced agglomeration of these NPs in solution. The average sizes of these magnetic nanoparticles are in the range required for superparamagnetism, as well as for biomedical applications.^63^

**Fig. 2:**
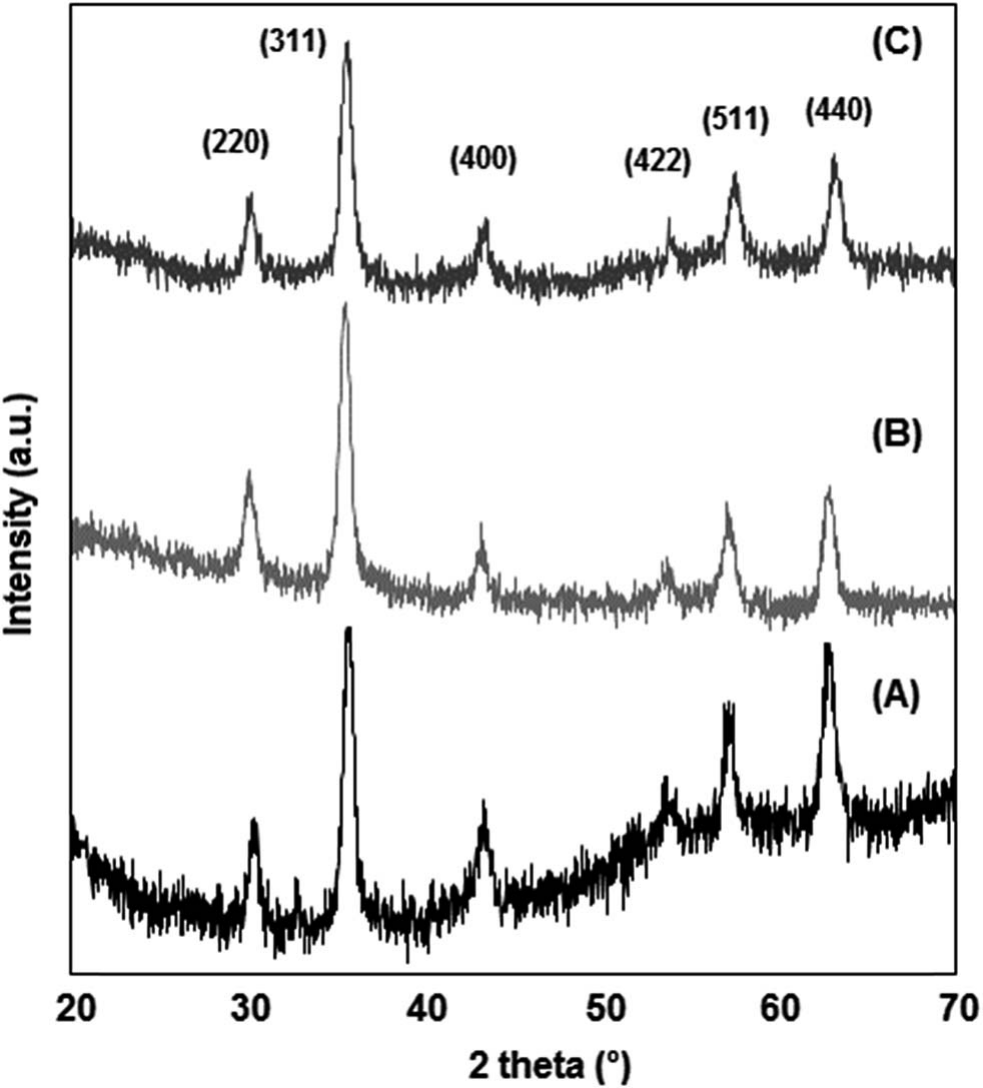
XRD pattern showing the characteristic magnetite/maghemite diffraction peaks of the prepared nanoparticles: (A) Fe_3_O_4_; (B) Fe_3_O_4_@CMC; (C) Si–Fe_3_O_4_@CMC.

**Fig. 3:**
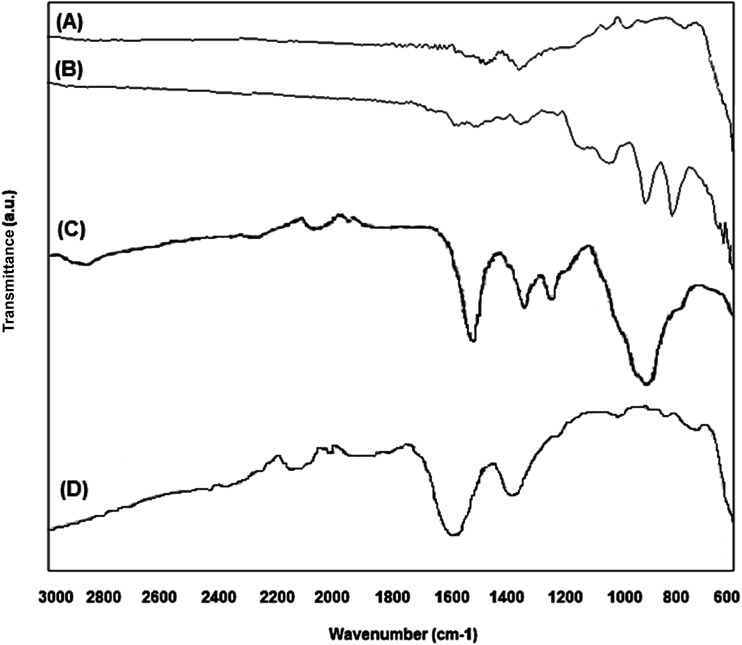
Fourier transform infrared spectra of the prepared magnetic nanoparticles: (A) Fe_3_O_4_; (B) Si–Fe_3_O_4_; (C) pure CMC; (D) Si–Fe_3_O_4_@CMC.

The nanoparticle's crystalline phase was identified by X-ray diffraction (XRD) (Fig. 2). The XRD pattern (Fig. 2) showed six characteristic peaks [(220), (311), (400), (422), (511), and (440)], which are consistent with the main phases of the magnetite and maghemite diffraction peaks, and no traces of other iron oxide phases were found. The silanization or CMC coating of magnetite nanoparticles did not influence the crystalline phases. Fourier transform infrared spectra of the prepared nanoparticles (Fig. 3A) show that the characteristic band for the Fe–O stretching was located near 590 cm^−1^. New vibrational bands appear at frequencies between 800 and 1100 cm^−1^ due to the presence of aminopropylsilane on the surface (Fig. 3B) and between 1000 and 1700 cm^−1^ associated with C–O and C–C bonds due to the presence of CMC on the nanoparticle surface (Fig. 3D). The polysaccharide molecules adsorbed on the nanoparticle surface stabilize the particles in aqueous suspension. The surface charge of the CMC coated magnetite NPs was in the range of −56 to −69 mV, as determined by zeta potential (*ζ*) measurements.

### Nanoparticle internalization and cytotoxicity

**Fig. 4:**
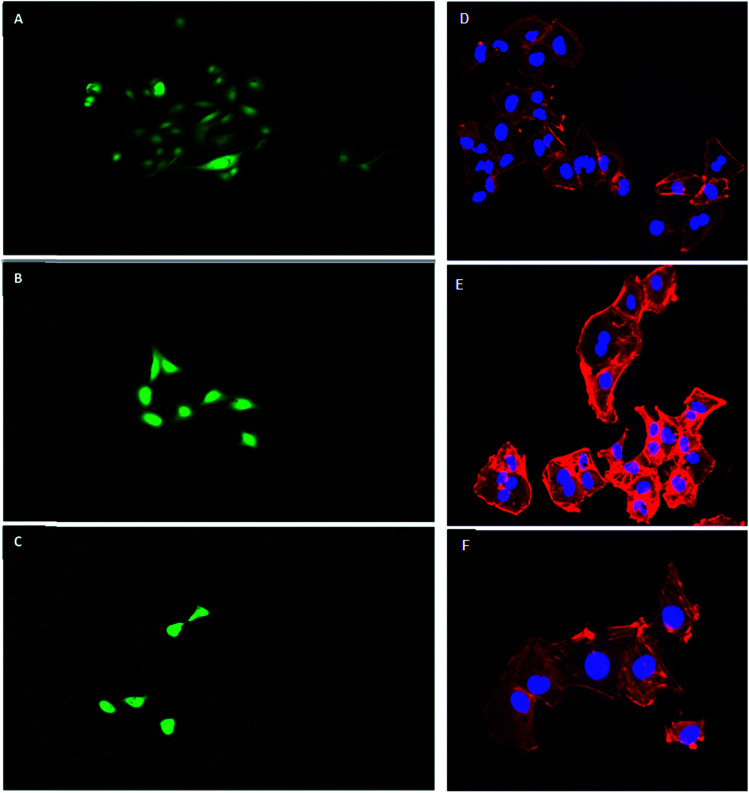
Fluorescence microscopy micrographs of HLMVE cells, after 24 h of exposure to 0.1 mg mL^−1^ of magnetic nanoparticles: cells stained with calcein-AM and ethidium homodimer-1: (A) Si–Fe_3_O_4_@CMC, 10×; (B) Fe_3_O_4_@CMC, 10×; (C) Fe_3_O_4_, 10×; and rhodamine phalloidin and DAPI stained cells, (D) Fe_3_O_4_, 10×; (E) Fe_3_O_4_@CMC, 10×; (F) Si–Fe_3_O_4_@CMC, 10×.

**Fig. 5:**
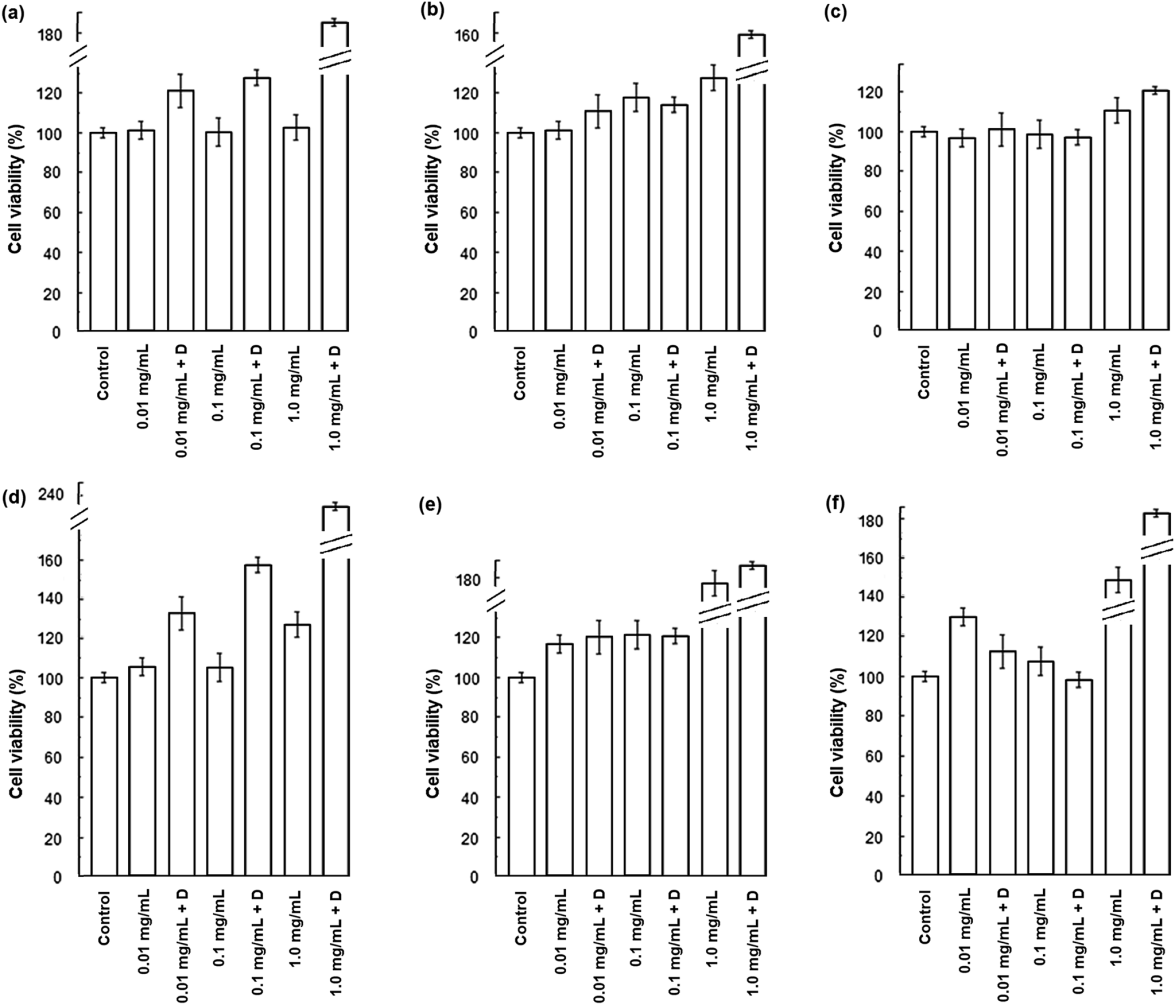
Cell viability for HLMVE cells exposed to 0.01, 0.1 and 1.0 mg mL^−1^ doses of magnetic nanoparticles, with and without dopamine (+D) loading, after 24 h: (a) Fe_3_O_4_; (b) Fe_3_O_4_@CMC; (c) Si–Fe_3_O_4_@CMC; and after 48 h: (d) Fe_3_O_4_; (e) Fe_3_O_4_@CMC; (f) Si–Fe_3_O_4_@CMC.

HLMVE cells were exposed to 0.1 mg mL^−1^ of magnetic nanoparticles in the cell culture medium for 24 h. Analysis by confocal fluorescence microscopy of cells stained with calcein AM and ethidium homodimer-1 showed isolated live cells (green), negligible aggregation of the nanoparticles and no evidence of dead cells (red) after 24 h of incubation (Fig. 4a–c). The analysis of HLMVE cells stained with rhodamine phalloidin and DAPI (Fig. 4d–f) showed isolated cells, with no morphological abnormalities of the cytoskeleton and well-formed nuclei after 24 h of exposure to the nanoparticles. These results indicate that the nanoparticles have no significant effect on the cell morphology or development, suggesting that they are not toxic to them. The degree of cell survival (cell viability) was evaluated by the standard methyl thiazol tetrazolium bromide (MTT) assay. The analysis of cytotoxicity after incubation of HLMVE cells with 0.01, 0.1 and 1.0 mg mL^−1^ of nanoparticles showed that the viability of cell cultures is not significantly affected or modified by the presence of the nanoparticles after 24 and 48 h of treatment (90–100% viability in relation to the control sample, as shown in Fig. 5a). No negative effect on cell viability was observed when the same systems (Fe_3_O_4_, Fe_3_O_4_@CMC, and Si–Fe_3_O_4_@CMC) were loaded with dopamine (Fig. 5b).

**Fig. 6:**
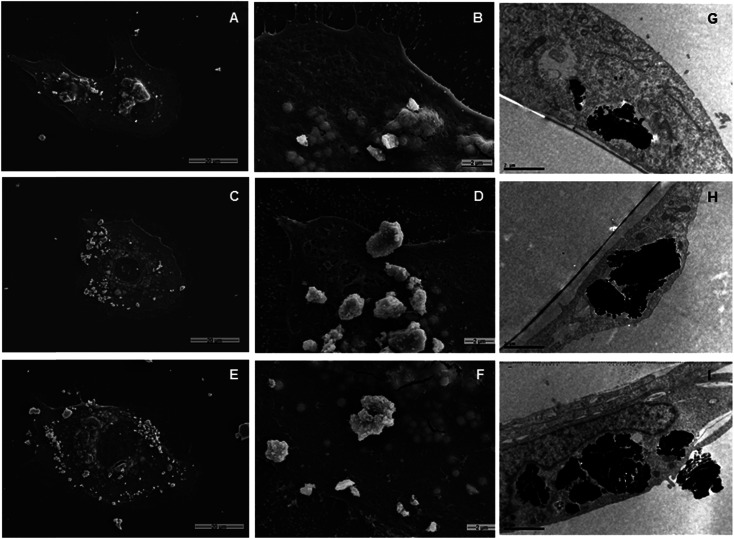
SEM and TEM micrographs of HLMVEC after 24 h of exposure to 0.1 mg mL^−1^ of nanoparticles; SEM: (A) Fe_3_O_4_, 1000×; (B) Fe_3_O_4_, 6000×; (C) Fe_3_O_4_@CMC, 1000×; (D) Fe_3_O_4_@CMC, 6000×; (E) Si–Fe_3_O_4_@CMC, 1000×; (F) Si–Fe_3_O_4_@CMC, 6000×; TEM: (G) Fe_3_O_4_; (H) Fe_3_O_4_@CMC; (I) Si–Fe_3_O_4_@CMC.

Both SEM and TEM analyses were used to examine the interaction of the nanoparticles with the cell membrane. Fig. 6a–c show overlapped nanoparticle aggregates and HLMVE cells. Although it is not clear whether the nanoparticles are on the cell membrane or inside, almost no nanoparticles were found “outside” the cells, and all cells presented external morphological changes (pseudopod-like) that may be related to an endocytosis or pinocytosis process during the interaction with the magnetic nanoparticles. In addition, a large number of actin fibrils (worm-like structures in Fig. 6d and f) were observed in the intracellular medium, which suggests that a cell internalization process took place. Considering that cells start assimilating nutrients and other substances from the culture medium during the first hour, the observation of large aggregates near the cell nucleus suggests that nanoparticles are internalized, although it is not clear what the uptake mechanism is. TEM analysis was performed to determine whether the nanoparticle aggregates were inside or outside the cells. Fig. 6g and h show slices of HMLVE cells that had been exposed to the loaded and unloaded magnetic nanoparticles. Large aggregates are visible inside one vesicle. No differences were observed in the aggregates of silanized or unsilanized magnetic nanoparticles coated with CMC. These results suggest that the nanoparticles, first, aggregate outside the cell membrane and then they are internalized into the cells through endocytosis or pinocytosis, accumulating inside specialized vesicles. The intracellular pattern of nanoparticle distribution in the cytoplasm, outside the cell nucleus, may indicate that they are stored inside endosomes, which are acidic compartments used for nutrient storage and digestion, suggesting that the mechanism of internalization is endocytosis. In previous studies, we have observed similar processes of nanoparticle uptake in different cell lines.^48,55^ The CMC coating increased the stability of both the silanized and unsilanized magnetic nanoparticles, thereby improving their chances of being internalized by the HMLVE cells.

### Transwell inset assay

An *in vitro* model consisting of a monolyaer of HLMVE cells was used for preliminary evaluation of the BBB permeability of the CMC coated magnetic nanoparticles. For this study, a bi-compartmentalized transwell was employed in which HLMVE cells were grown to confluence in the upper chamber. HLMVE cells were seeded onto transwell inserts on 24-well plates (*n* = 2) containing Ham's F-10 medium at 37 °C and 5% CO_2_ (1 × 10^5^ cells per well). The cells were incubated for 24 h, after this time the medium was replaced with fresh medium containing 0.1 mg mL^−1^ of fluorescein-labeled Fe_3_O_4_@CMC or Si–Fe_3_O_4_@CMC nanoparticles.

**Fig. 7:**
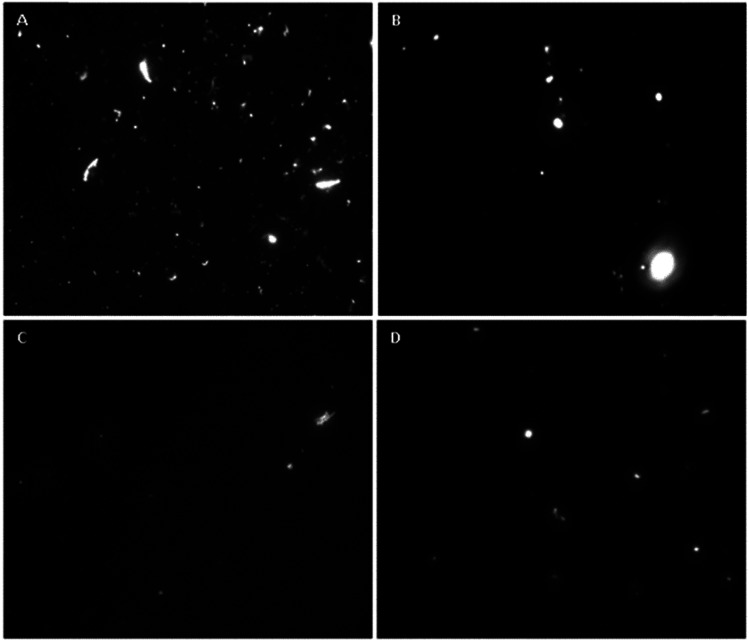
Fluorescence microscopy images in Ham's F-10 medium recovered from the lower chamber of transwell inserts with a monolayer of HLMV endothelial cells after 24 and 48 h of exposure to fluorescein-labeled magnetic nanoparticles: (A) Si–Fe_3_O_4_@CMC after 24 h; (B) Si–Fe_3_O_4_@CMC after 48 h; (C) Fe_3_O_4_@CMC after 24 h; (D) Fe_3_O_4_@CMC after 48 h.

The integrity of the grown BBB model was evaluated using TEER measurements at the onset and during the experiments, and these were compared to the control (non-cultured wells). Before the addition of fluorescein-labeled magnetic nanoparticles, the TEER value for the non-cultured wells (control) was on average 219 ± 7 Ω cm^2^. 24 h after the addition of magnetic nanoparticles the TEER values of Fe_3_O_4_@CMC and Si–Fe_3_O_4_@CMC were on average 212 ± 5 Ω cm^2^ and 195 ± 5 Ω cm^2^, respectively, while after 48 h they were on average 197 ± 13 Ω cm^2^ and 185 ± 12 Ω cm^2^. The resulting TEER value of ∼200 Ω cm^2^ is considered consistent with the formation of an intact BBB. Aliquots were taken from the lower chamber after 24 and 48 h and analyzed with a fluorescence microscope. Fluorescein labeling of the magnetic nanoparticles was used for monitoring their transmigration from the upper to lower chamber in the transwell inset through the BBB model. In Fig. 7, the presence of fluorescent nanoparticles is evident in the recovered medium of the lower chamber for both systems (fluorescein labeled Fe_3_O_4_@CMC and Si–Fe_3_O_4_@CMC), even after just 24 h. This result indicates that the fluorescein labeled, CMC coated magnetic nanoparticles can cross through the densely packed barrier of HLMV endothelial cells used as a BBB model. Silanized magnetite nanoparticles coated with CMC had a higher transmigration rate than those that were not silanized, and they had a lower propensity for aggregation in the medium. The values of TEER were near the standard 200 Ω cm^2^ at the beginning of the experimental period and remained close to this value after 24 and 48 h, suggesting that exposure to the fluorescein labeled, CMC coated magnetic nanoparticles did not compromise the integrity of the BBB model. The results obtained from this BBB model are in agreement with those previously obtained by Thomsen and co-workers, who studied the uptake and transport of SPIONs through an *in vitro* BBB model made of human brain capillary endothelial cells (HBCEC).^64^

### *In vitro* drug loading and release of the magnetic nanoparticles

**Fig. 8:**
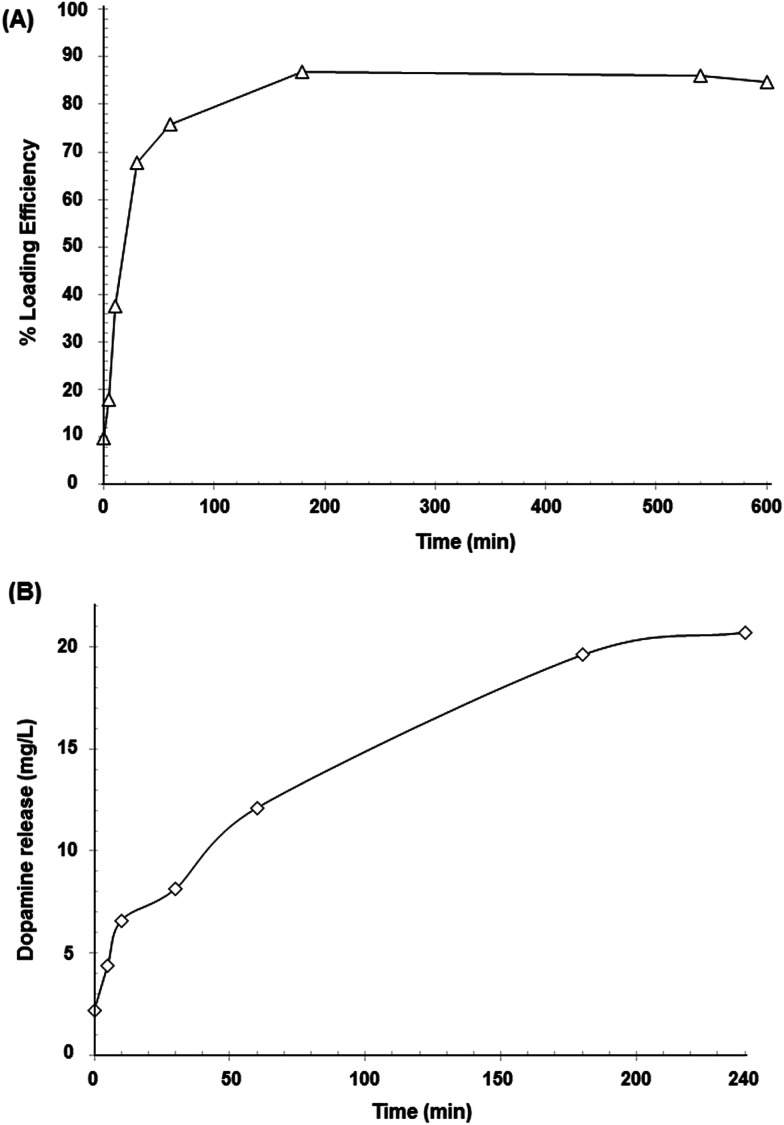
Dopamine profiles for (A) drug LE% and (B) drug release (mg L^−1^) in aqueous solution at room temperature and pH 7.

The Fe_3_O_4_@CMC system was selected for the evaluation of dopamine loading and release. For this evaluation, magnetic nanoparticles were loaded with dopamine. Up to 0.13 mg of dopamine was loaded in the nanocarrier per mg of magnetite. This amount was calculated by considering the total amount of dopamine available in the solution and the amount of magnetite dispersed in it. Fig. 6 shows the drug loading efficiency percentage (LE%) for Fe_3_O_4_@CMC over a period of 10 h and the dopamine release profile over 4 h at room temperature and pH 7.0. A drug LE% of 84.6% was estimated (Fig. 8a), which is comparable with those of other drug-loaded drug nanocarriers reported in the literature.^65^ These results indicate that the CMC coated Fe_3_O_4_ nanoparticles load up to 70% of the available drug during the first hour and then, they can release up to 81.6% of the loaded dopamine when re-dispersed in DI water. Fig. 8b shows the cumulative amount of released dopamine from the magnetic nanocarrier *versus* time. Dopamine release occurred rapidly during the first hour (nearly 40%); this was likely to have been the dopamine adsorbed on the surface of the carrier. The remaining dopamine released at a slower rate during the next few hours, which can be associated with the release of the dopamine in the polymeric coating, reaching almost an invariant concentration in solution after 4 hours. The sustained-release pattern suggests that this system may be a good candidate for controlled drug delivery and release, although the release profile in physiological solution still needs to be determined.

Dopamine release kinetics were analyzed using various mathematical models (zero-order, *Q*_*t*_ = *Q*_0_ + *K*_0_*t*; first order, ln *Q*_*t*_ = ln *Q*_0_ + *K*_e_*t* and Higuchi, *Q*_*t*_ = *Q*_0_ + *K*_h_*t*^1/2^). Considering the *R*^2^ values, the calculated zero-order (*R*^2^ = 0.926) and first-order (*R*^2^ = 0.718) models were not appropriate to describe the drug release kinetics. The release kinetics appeared to be square root time-dependent, as in the Higuchi model (*R*^2^ = 0.992), suggesting that diffusion plays an important role in the release of the drug.

CMC-coated magnetic particles appear to have significant potential for *in vivo* magnetic field assisted drug delivery. As magnetic ferrofluids are capable of interaction with external magnetic fields, these colloidal dispersions are ideal for magnetic targeting drug delivery applications. The use of a magnetic ferrite core gives Fe_3_O_4_@CMC NPs the potential to be actively delivered, localized, and targeted to the desired region, tissue or type of cell. After the administration of these SPIONs, an external magnetic field may be applied to the region of interest, drawing the NPs to that specific site.^66^ The null residual magnetization of SPIONs after removal of the external magnetic field allows the NPs to disperse and avoids magnetically induced agglomeration in veins or arteries and also avoids phagocytosis while the circulation half-time is increased.^46,67^ Stereo-specific manipulation of magnetic fields to guide nanoparticles to a precise location in the brain is currently being studied.^68^ Nevertheless, there are still many challenges to overcome before considering clinical applications of this technology for brain targeting in humans. Studies using the application of static, strong Nd–Fe-B magnets to certain anatomic regions showed large concentrations of SPIONs in tissues close to the permanent magnet, with a decrease in concentration as the distance from the magnet increases, clearly indicating that the NP migration in the body can be controlled by using external magnetic fields.^69–71^ However, studies have shown that it is more difficult to target sites located farther from the magnetic source and specific targeting has been more successful in small animal models than in larger animals or humans. Technological advances such as the development of devices capable of generating “on/off” magnetic fields within the brain would optimize the practical application of magnetically targeted drug delivery systems for the treatment of neurological disorders.

The surface charge of CMC coated magnetite NPs is dependent on the pH and the amount of CMC and may be tailored to improve their BBB permeability. Although studies have shown that NPs with high positive charge are immediately toxic to the BBB,^72^ in some cases NPs with moderate (−1 to −15 mV) or high (−15 to −45 mV) negative zeta (*ζ*) potentials and even moderate (up to 15 mV) or higher positive *ζ*-potentials^73–76^ have been able to cross the BBB and deliver drugs to the brain.^77,78^ This may be due to the greater cellular uptake profile of positively charged NPs in brain microvessel endothelial cells compared with negatively charged IONPs of similar size.^79,80^ Experiments using magnetic field assisted permeability show increased uptake for nanoparticles with a greater negative charge, suggesting that negatively charged NPs are likely to follow a paracellular route, which makes them more suitable for magnetic assisted drug targeting.^81^ An *in vitro* study of carboxymethyl dextran-coated NPs concludes that the uptake of most negatively charged particles seems to occur *via* non-specific interactions.^39^

The characteristics of these versatile NPs make them apt for multi-modal functions. In addition to their potential for drug delivery to the brain, they can serve simultaneously as magnetic resonance imaging contrast agents. Furthermore, radiocontrast agents may be attached to these delivery systems, which would facilitate better imaging and diagnosis of neurodegenerative disorders. The negative *ζ*-potentials of CMC/Fe_3_O_4_ NPs make these particles apt for another interesting application for brain-targeted magnetic nanoparticles. As recently reported by Dante *et al.*, the NP surface charge is key for the modulation of neuronal electrical activity.^82^ Their findings regarding selective NP–neuron interactions open up the possibility for novel applications of NPs in neuroscience, specifically for the design of NPs capable of neuronal subtype-specific targeting. This research suggests that negatively charged NPs could be used in long-term imaging as markers of active neurons, which would enable visualization of the aberrant increased neuronal activity of neurological disorders. In addition, the increase in neuronal activity produced by these NPs could be used to increase the activity of inhibitory neurons with reduced excitability, which is a hallmark of the severe forms of epilepsy.^83^ These NPs may eventually be utilized to modulate the balance between excitation and inhibition in the brain, which is a significant factor in most neurological diseases.

## Conclusions

Superparamagnetic nanoparticles coated with a layer of the biocompatible polysaccharide CMC were prepared and fully characterized. Cell viability assays, as well as a complete analysis of their interaction with the cell membrane, cell internalization and ability to pass through a model of the blood-brain barrier (BBB), were performed in cell cultures. No indication of toxicity was found, and our results indicated that the nanoparticles accumulated in endosomes. Clear evidence of crossing through a barrier of densely packed endothelial cells was observed; these results indicate that CMC coated magnetic nanoparticles may show great potential as drug delivery systems for neurological treatments. Further testing is required for the continued development of this drug delivery system.

In summary, the physicochemical properties of CMC/Fe_3_O_4_ NPs can be tailored for use in different applications and are very attractive for brain-targeted magnetic nanoparticle research. After the attachment of drug molecules to these delivery systems, the neutral to negative surface charge of the CMC coating may facilitate BBB crossing.^82,84^ The biodegradability of the coating of the nanoparticles allows the drug to be delivered at a controlled and sustained rate to the target site in the brain. In addition to the already proven advantages of paramagnetic NPs for drug delivery, the ferrite cores allow magnetic field targeting that could favor both permeability of the BBB and specificity of the drug release site.^64,85^ The CMC coating creates a hydrophilic surface that avoids agglomeration, increases nanoparticle dispersion in physiological solution and extends bioavailability; it also provides an appropriate surface for functionalization with targeting moieties to trigger drug release or an enzymatic stimulus, which would allow for even more precise targeting of specific cells. Targeted delivery of these systems would significantly reduce the required dosage of the therapeutic agent, which may decrease the undesired effects of the treatment of neurological disorders such as adverse reactions and toxicity. Furthermore, the simplicity of preparation, plentiful supply and the safety and biocompatibility of the components of these CMC coated magnetite NPs could offer a considerable reduction in the cost of the developmental phase of brain-targeted pharmaceuticals using these delivery systems.

## Author contributions

The manuscript was written with the contribution of all authors. All authors approved the final version of the manuscript.

## Conflicts of interest

All authors declare no competing interest.

## Supplementary Material
